# The immunogenic potential of AZD1402 (Elarekibep) T-cell epitopes in healthy volunteers and drug-exposed clinical trial participants

**DOI:** 10.1007/s00204-026-04403-1

**Published:** 2026-04-28

**Authors:** Liam Farrell, Stephanie M Bates, Alexander Walker, Monday Ogese, Xiaoli Meng, Sian Piper, John Mo, Katy Saide, Jorrit Hornberg, David Close, Ian Wallace, Catherine J Betts, Dean J Naisbitt

**Affiliations:** 1 Centre for Drug Safety Science, Department of Pharmacology and Therapeutics, University of Liverpool, Liverpool, UK; 2 Safety Sciences, Clinical Pharmacology & Safety Sciences, AstraZeneca R&D, Cambridge, UK; 3 Respiratory and Immunology, AstraZeneca R&D, Cambridge, UK; 4 Chief Medical Office, Research & Development, Patient Safety Biopharma AstraZeneca, Gothenburg, Sweden; 5 Safety Sciences, Clinical Pharmacology & Safety Sciences, AstraZeneca R&D, Gothenburg, Sweden; 6 Early Clinical Development, Research and Development, AstraZeneca R&D, Cambridge, UK

**Keywords:** Anticalin, Human, Hypersensitivity, Immune-mediated adverse event, T-cells

## Abstract

AZD1402 (Elarekibep) is a novel anticalin with engineered IL-4Rα antagonist binding properties that was under development for inhaled treatment of asthma. Structural similarity to tear-lipocalin is thought to limit immunogenicity risk; however, a range of treatment-emergent hypersensitivity-like adverse events were observed in a clinical study. This study aimed to (i) explore the activation of drug-exposed human T-cells with AZD1402- and tear lipocalin-derived peptides, (ii) characterise any T-cell epitopes contained within AZD1402 and (iii) define the nature of any response. Peripheral blood mononuclear cells (PBMC) from AZD1402-exposed study participants, alongside AZD1402-naïve donors, were cultured with AZD1402 and AZD1402-derived overlapping 18mer peptides. Proliferation and cytokine secretion were measured. T-cell clones were generated to characterise the diversity of T-cell epitopes and reactivity against equivalent tear-lipocalin peptides. AZD1402 contained multiple epitopes located in engineered “hotspot” regions of the molecule that activated PBMC and MHC class II-restricted CD4 + T-cell clones from both study participants and AZD1402-naïve donors. T-cell stimulatory peptides contained amino acid substitutions generated through mutagenesis to create AZD1402 target binding properties; similar T-cell epitopes were not found in tear-lipocalin. Detection of T-cell clones responsive towards AZD1402 and AZD1402-derived peptides provides evidence that immunogenic epitopes are formed naturally through processing of the AZD1402. These data describe a recall immunogenicity risk associated with AZD1402 and provide a framework by which to explore the immunogenic potential of modified sequences within anticalin or other large molecule therapeutics.

**Supplementary Information:**

The online version contains supplementary material available at 10.1007/s00204-026-04403-1.

## Introduction

Lipocalins are a family of low molecular weight (16–20 kDa) binding proteins that transport, store, or sequester small biological compounds like vitamins and hormones in many organisms. The highly conserved core structure with 4 variable loops permits target binding; the loops differ in terms of length, amino acid sequence and backbone conformation, which explains the broad spectrum of ligand specificities (Schiefner et al. 2015).

**Fig. 1:**
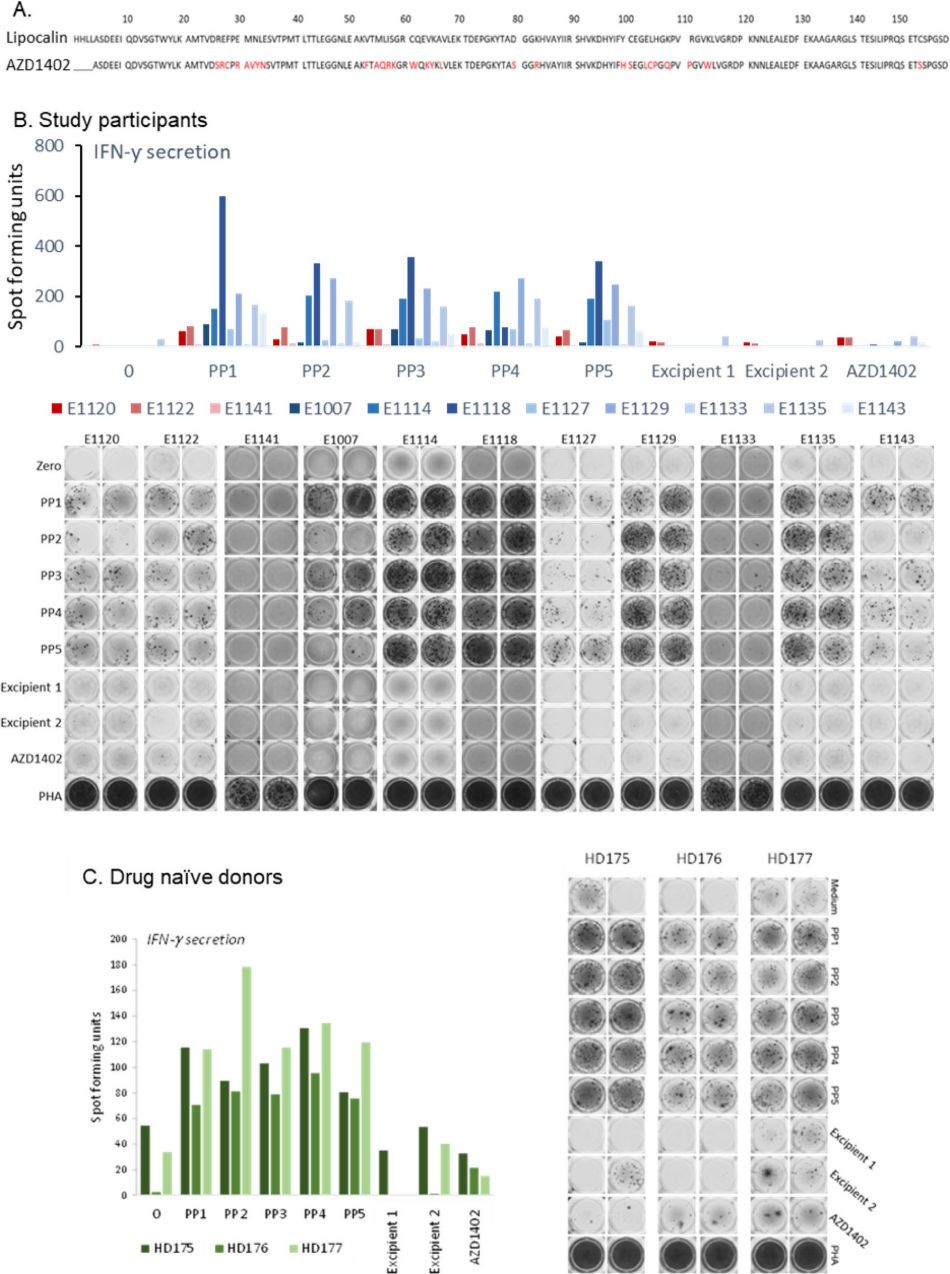
ELISpot analysis of IFN-γ secretion from PBMC of **A** AZD1402 study participants and **B** non-exposed individuals. PBMC were incubated in an ELISpot plate, pre-coated for IFN-γ, in the presence of PP1-5, excipients 1–2 (Ex1 & Ex2), and AZD1402 at the working concentrations: peptide pools (10µM), excipients (50µM) or AZD1402 (100µM) for a period of 48 h with R9 medium as a negative control and PHA as a positive control. Data are reported as spot forming units with quantitative data (mean of duplicate wells) and images of the wells shown. Study participants in red are those which exhibited adverse events

In recent years the ligand-binding site of natural lipocalins have been subjected to modification via combinatorial protein engineering whereby libraries of mutations are generated and those with increased target binding and/or novel binding functions are selected. Anticalin proteins derived from this process are homologous with naturally occurring lipocalins (Deuschle et al. 2021; Gille et al. 2016; Rothe and Skerra 2018; Vogt and Skerra 2004) with a similar overall size as the lipocalin upon which they are based, due to the design procedure being limited to amino acid exchanges without insertions or deletions to retain the secondary and tertiary structure of the molecule (Achatz et al. 2022; Schönfeld et al. 2009). The small size of the protein is thought to make anticalins an ideal drug platform for the inhaled dosing route. AZD1402 (Elarekibep), a potent and selective antagonist of IL-4Rα developed as a potential new medication for asthma, is derived from and has structural similarity to endogenous human tear lipocalin (Lcn1; also known as lipocalin-1). AZD1402 is 158 amino acids in length with 28-point mutations from the original protein, 26 of which are located within the variable loops and hence influence the target binding site of the molecule (Fig. 1a) (Matschiner et al. 2023). In a clinical pharmacokinetic study, different formulations were administered in random order in a cross-over design, at least 5 days apart, to compare a nebuliser solution with that of dry powder. Of the 18 healthy volunteers, 5 experienced treatment emergent adverse events that started in the hours following the third administration (https://clinicaltrials.gov/study/NCT03921268). Due to the proximity to dosing of the treatment-related adverse events and evidence of increased C-reactive protein, the clinical picture was consistent with a hypersensitivity-like event occurring in response to administration of AZD1402. Findings in a preclinical toxicity study resulted in discontinuation of both the phase 2a trial with AZD1402 and the development program. Since immunogenicity involves the presentation of MHC class II associated peptides (formed through antigen presenting cell processing of the therapeutic protein) to CD4 + T-cells (Attermann et al. 2021; Barra et al. 2020; Jawa et al. 2013; Moussa et al. 2016; Sauna et al. 2020; Sivelle et al. 2023), this study was designed to investigate whether T-cell responses against the full AZD1402 protein or AZD1402-derived peptides were detectable in study participants with and without adverse events. The study also aimed explore the diversity of T-cell epitopes and T-cell reactivity against equivalent tear lipocalin peptides.

## Methods

### Study participants and ethical implications

The trials unit Parexel International were incorporated as a recruitment site onto Liverpool University NHS ethics (A mechanistic investigation into drug and chemical induced hypersensitivity [HYST] SA13). Of the 18 pharmacokinetic bridging study participants (https://clinicaltrials.gov/study/NCT03921268), 11, including 3/5 (E1120, E1122, E1141) who experienced adverse effects, were recruited to this study (Table 1) alongside AZD1402-naive donors from Liverpool. All subjects were informed orally about the study and individuals each signed the corresponding informed consent form. Healthy donors were recruited from the Liverpool Pharmacology Biobank under ethical approval from the Liverpool Research Ethics Committee. All donors gave written and informed consent according to the Declaration of Helsinki and were treatment naïve for AZD1402. Up to 108 mL of venous blood was drawn into lithium heparin coated tubes (Greiner Bio-One) to study T-cell responses. Due to the SARS-Cov2 pandemic and restricted access to individuals for extensive periods, blood samples were collected from the 11 pharmacokinetic bridge study participants approximately 18 months following clinical exposure to AZD1402. Genomic DNA was extracted from PBMC from the 11 pharmacokinetic study participants and naïve donors using Chemagic magnetic separation (Chemagen, Baesweiler, Germany). High-resolution sequence-based HLA typing using next generation sequencing platforms was performed by Histogenetics laboratory (Histogenetics, New York, USA) at the following 6 loci: HLA-A, -B, -C, -DRB1, -DPB1 and -DQB1.

### Reagents

AZD1402 and formulation excipients 1 (D-(+)-Trehalose Dihydride) and 2 (L-leucine) were kindly supplied by AstraZeneca (Gothenburg, Sweden) at a stock concentration of 3.14mM. Tear lipocalin was a supplied by Pieris Pharmaceuticals (Munich, Germany) at a stock concentration of 700µM. Proteins were aliquoted and stored at -80 °C until use. All peptides (≥ 95% pure) were purchased from SynPeptide (Shanghai, China) and reconstituted in DMSO at a stock concentration of 25-50mM, aliquoted and stored at -20 °C until use. The 69 AZD1402-derived 18mer peptides were created from sequences stepped by 2aa spanning the full length of AZD1402. Pools of AZD1402-derived peptides (PP1-5) were generated containing 12–16 peptides per pool for initial PBMC analysis (Supplementary Table 1). For characterisation and the generation of T-cell clones, a pool was created comprising a selection of 12 PBMC stimulatory AZD1402-derived peptides (nos: 9, 10, 19, 20, 21, 26, 30, 43, 44, 47, 48 and 50) and referred to as the primary peptide pool (PPP). Tear lipocalin-derived peptides were generated from the correspondingly positioned wild type sequences to control for the AZD1402-derived peptides present in the PPP.

### Peripheral blood mononuclear cell isolation

Venous blood samples were transported to Liverpool for immediate peripheral blood mononuclear cell (PBMC) isolation. Whole blood was layered onto Lymphoprep (Stemcell technologies, Cambridge, UK) and centrifuged at 800 g for 20 min to separate the individual blood cellular components. The cloudy layer containing PBMC was harvested and washed using phosphate buffered saline prior to cell counting using a haemocytometer. Fresh PBMC were used for the lymphocyte transformation test and PBMC ELISpot as detailed below. Remaining PBMC were cryopreserved (80% foetal bovine serum, 20% DMSO) and stored in a -150 °C Human Biological Sample-compliant freezer until subsequent analysis.

### Cell culture medium

Cell culture medium for T-cells (R9) is composed of RPMI 1640 supplemented with 5% heat-inactivated human AB serum, HEPES (25mM), penicillin (1000U/mL), streptomycin (0.1 mg/mL), L-glutamine (2mM) and transferrin (25 µg/mL). EBV transformed B-cells were cultured in F1 medium composed of RPMI supplemented with 10% foetal bovine serum, HEPES (25mM), penicillin (1000U/mL), streptomycin (0.1 mg/mL) and L-glutamine (2mM).

### PBMC proliferation inhibition assay

PBMC (1.5 × 10^5^/100 µL) obtained from 7 AZD1402-naive donors (HD175, HD176, HD176, HD755, HD756, HD757 and HD762) were incubated with AZD1402 or excipient 1 and 2 for 24 h. Phytohemagglutinin (PHA) was added at 10 µg/mL and cells were incubated for a period of 2 days with [^3^H]thymidine (0.5µCi; Moravek Biochemicals Inc, Brea, CA) added for the final 16 h. Wells were harvested onto filter mats and proliferation was measured using scintillation counting. The objective of this experiment was to obtain the maximum concentration of test compounds tolerated by PBMC. We acknowledge the use of radiometric readouts in this study may be prohibitive for use as an immunogenicity screening platform at large. Alternative proliferation readouts and/or cytokine secretion can be used in place of [^3^H]-thymidine incorporation in all experiments herein.

### Lymphocyte transformation test

PBMC (1.5 × 10^5^/100µl) were incubated with non-toxic concentrations of peptides, PP1-5 (as described in reagents list), excipient 1 and 2, and full proteins (AZD1402 and tear lipocalin). Culture media was used as a negative control while PHA (10 µg/mL) was used as a positive control. Cells were incubated for a period of 6 days with tritiated thymidine (0.5µCi) added for the final 16 h. Proliferation was measured using scintillation counting as above. Supernatant (50µL), collected prior to the addition of [^3^H] thymidine, was stored at -80 °C until cytokine assessment.

### PBMC cytokine secretion

ELISpot plates were activated with 15µL of 35% EtOH and washed five times with dH_2_O. The plates were then coated with 100µL/well of interferon gamma (IFN-γ) capture antibodies (15 µg/mL) and incubated overnight at 4 °C. The following day, wells were washed five times with sterile PBS and then blocked with 200µL of R9 medium at room temperature for a period of 30 min. PBMCs (5 × 10^5^/100µL) were incubated in the presence of peptides, PP1-5, excipients, and full proteins at various concentrations for 48 h. R9 and PHA (10 µg/mL) were used as the negative and positive controls, respectively. Following incubation, the ELISpot plate was developed according to the manufacturer’s instructions.

Supernatants were subjected to multiplex bead array analysis (Milliplex human cytokine panel, HCYTOMAG-60 K) as per manufacturer’s instructions (Magpix Bead array analyser, Millipore, Watford, England). The analysis was performed with supernatants from 8 study participants with available PBMCs and a representative 5 individual AZD1402-naive donors.

### Activation of CD3- and CD45RO-depeleted PBMC with AZD1402

PBMC were depleted of CD3 + T-cells and CD45RO + T-cells by positive selection using microbeads Miltenyi magnetic LS-columns. The procedure was repeated on multiple occasions to obtain greater than 97% depletion. The CD3- and CD45RO-depleted PBMC were then used in the lymphocyte transformation test/IFN-γ ELISpot with AZD1402 and AZD-1402-derived peptides as described above. Flow cytometry was performed using CD3-PerCP-Cy5.5 (clone UCHL1, Biolegend, UK) and CD45RO-PE (clone SK7, Biolegend, UK) fluorescent antibodies. Total PBMC, CD3- depleted cells and CD45RO- depleted cells (all 1 × 10^5^) were stained and samples were analysed by flow cytometry (FACSCanto II, BD Life Sciences, USA). A total of 10,000 events were acquired for each sample. Percentage of positive and negative populations were calculated using predefined gates to confirm approximately 3% of CD3 and CD45RO- cells remaining in depleted conditions.

### Generation of antigen-responsive T-cell clones

Antigen-enriched T-cell lines were generated via the culture of PBMC with either AZD1402 or a pool of PBMC stimulatory AZD1402-derived peptides (referred to as the PPP, Fig. 4c) for a period of 14 days. IL-2 (50IU/mL) was added on days 6 and 9 to maintain cellular proliferation. On day 14 T-cells were cloned by means of serial dilution, with cells being seeded at 3, 1, 0.3 cells per well (minimum of 3 96-well plates/condition) with 100µL of stimulatory cocktail containing IL-2 (200IU/mL), PHA (5 µg/mL), and irradiated allogenic PBMC (as feeder cells; 0.5 × 10^6^/mL). Cells were cultured for 5 days then fed using R9 and IL-2 (25IU/mL) every 2 days. Fourteen days after serial dilution T-cells were restimulated using 50µL of a stimulatory cocktail containing IL-2 (50IU/mL), PHA (5 µg/mL) and irradiated PBMC (1 × 10^6^/mL). Approximately 14 days after restimulation expanded clones were selected, further expanded and assessed for specificity. The specificity of T-cell clones was ascertained by culturing T-cell clones (5 × 10^4^/50µL) with autologous EBV-transformed B-cells (1 × 10^4^/50µL) in the presence and absence of AZD1402 or PPP for a period of 48 h. Following incubation, tritiated thymidine (0.5µCi) was added for a further 16 h and cellular proliferation was measured via scintillation counting. Clones which yielded a stimulation index of 2 or greater were subjected to mitogenic expansion in the presence of IL-2 for a period of 14 days prior to assessment of phenotype and functionality.

### Characterisation of AZD1402 and AZD1402-derived peptide-responsive T-cell clones

Specificity of T-cell clones against titrated concentrations of the PPP, individual peptides, lipocalin-derived peptides, AZD1402 or tear lipocalin was confirmed via the co-incubation of the T-cell clones (5 × 10^4^/well) with autologous EBV transformed B-cells (1 × 10^4^/well) for a period of 48 h (37 °C, 5% CO_2_). T-cell reactivity was ascertained via quantification of proliferation or IFN-γ secretion. The cellular phenotype of T-cell clones was determined by flow cytometry (FACS Canto II, BD) with CD4-FITC & CD8-PE fluorescent antibodies (BD Pharmingen, San Jose, USA). A total of 10,000 events were acquired for each sample. The profiles of secreted IFN-γ, IL-13 and granzyme B from AZD1402 and PPP-responsive clones were measured by Fluorospot according to manufacturer instructions (Mabtech, Nacka Strand, Sweden). T-cell clones and EBV-transformed B cells were also pre-treated with either isotype (IgG1) or HLA class-I (DX17), HLA class-II (Tu39) or HLA-DR (G46-6) blocking antibodies (all at a final concentration of 10 µg/mL, purchased from BD Pharmingen, San Jose, USA) for 2 h before the addition of optimal concentrations of AZD1402 or the PPP (100µM or 10µM, respectively).

## Results

### Adverse events show no clear association with HLA carriage

No clear association between HLA allele carriage and adverse event was detected, albeit with a very low number of study participants that developed adverse events (*n* = 3; Table 1) HLA-DP1*04:01 was expressed by all 3 reaction participants; however, the frequency of the HLA-DP1*04:01 allele is 40–50% in European populations. Specific HLA class I alleles were observed in 2 of 3 study participants with adverse hypersensitivity-like events (e.g., A*021:01, C*06:02); however, the same alleles were also detected in participants that showed no adverse events.

**Table 1:**
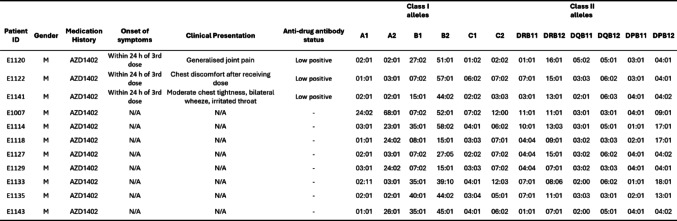
HLA typing and demographics of the study participants

### Inhibition assays to establish optimal compound concentrations for PBMC stimulation studies

AZD1402 inhibited PHA-induced PBMC proliferation at concentrations above 100µM (results not shown); however, viable PBMC remained at concentrations up to 800µM. Excipient 1 (D-(+)-Trehalose Dihydride) did not inhibit PHA-induced proliferation (1-1000µM), while excipient 2 (L-Leucine) inhibited proliferation at 100µM and above. Therefore, maximum working concentrations of 800µM, 1000 µM and 100 µM were used for AZD1402, excipient 1 and excipient 2, respectively. AZD1402 overlapping peptide pools (PP1-5, Supplementary Table 1) were used at non-inhibitory concentrations of 10µM and below.

### PBMC from pharmacokinetic study participants and non-exposed individuals proliferate in the presence of AZD1402-derived peptides

PP1-5 induced varying levels of proliferative responses from study participant PBMC (Supplementary Fig. 1). No clear differences in proliferation were noted between study participants reported with and without adverse events. PP1, 3 and 4 stimulated PBMC from at least 5 study participants to proliferate vigorously in a concentration-dependent manner. The magnitude of the induced proliferative response and number of responding pharmacokinetic study participants was lower with PP2 and PP5. Upon culture with the full protein, PBMC from 3 donors (E1122, E1135 and E1114) demonstrated proliferation in a concentration-dependent manner. The reduction in proliferation with the highest concentration of AZD1402 for two of the donors is likely due to direct toxicity. Increased proliferation was not observed when study participant PBMC were cultured with excipients 1 or 2 alone.

PBMC from 1 of 3 AZD1402-naive donors (HD177) were stimulated to proliferate in the presence of PP1-5, with a concentration-dependent response observed with most peptide pools (Supplementary Fig. 1). Weaker peptide pool-specific proliferative responses were detected from the remaining two AZD1402-naïve donors (HD175 and HD176). AZD1402 stimulated proliferation of PBMC from donor HD175 at certain concentrations, but no clear concentration-response was discernible. The excipients did not stimulate PBMC proliferation, except for donor HD177, where a minor increase at the highest concentration tested was noted.

### PBMC secrete IFN-γ in the presence of AZD1402-derived peptides

The majority of AZD1402 pharmacokinetic study participant PBMC were stimulated to secrete IFN-γ in the presence of PP1-5 (Fig. 1b). Donors E1133 and E1141 were the exceptions, where the level of IFN-γ secretion observed following culture with the peptide pools was similar to that noted for vehicle control wells. The levels of IFN-γ secreted by PBMC cultured with PP1, 3, 4 and 5 were similar, with lower levels of IFN-γ detected with PP2 in several study participants. No clear differences were noted between study participants with (E1120, E1122 and E1141) and without adverse events. PBMC secretion of IFN-γ was not detected with the excipients. Low levels of IFN-γ were secreted by PBMC from study participants E1120 and E1122 (both exhibited adverse events) exposed to the full protein AZD1402. The responses detected against AZD1402 full protein for the remaining trial participants and AZD1402 non-exposed donors were low. Similar to responses observed with PBMC from clinical study participants, IFN-γ secretion was detected from AZD1402-naive donor PBMC exposed to PP1-5 (Fig. 1c). The levels of IFN-γ secreted from PBMC from all study participants and naïve donors exposed to excipients were similar to control levels.

### PBMC secrete Th1 cytokines and cytolytic molecules in the presence of AZD1402-derived peptides

**Fig. 2:**
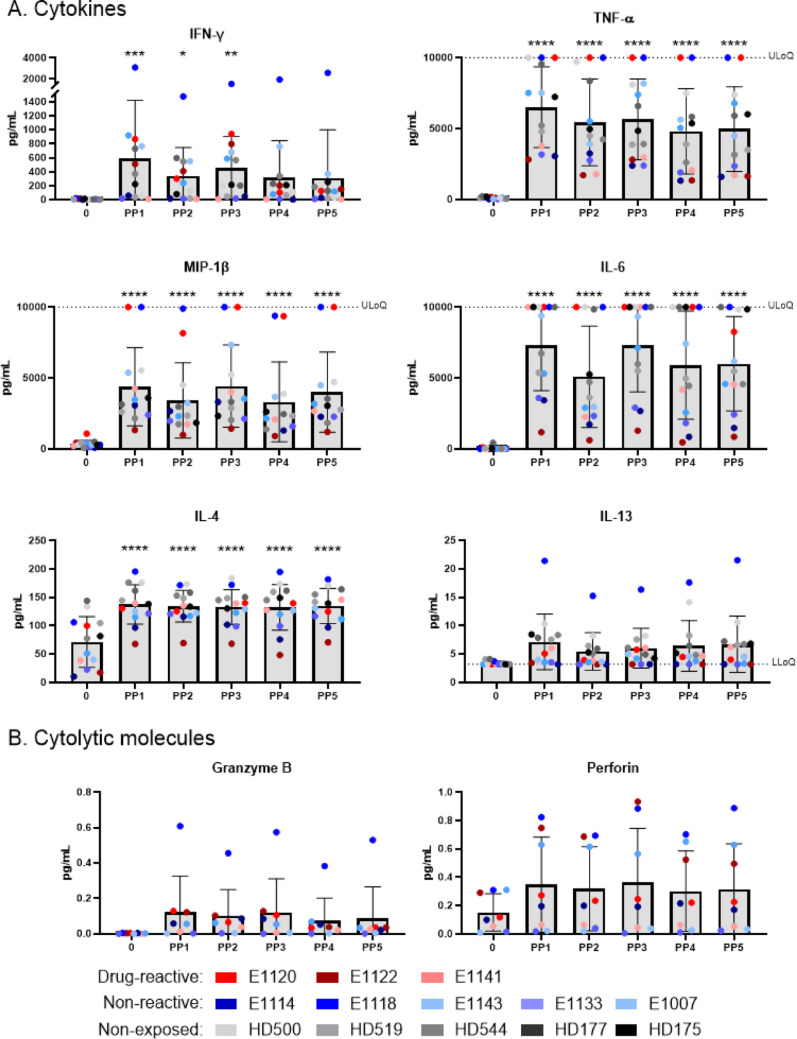
**A** Cytokine and **B** cytolytic molecule secretion from peptide pool-exposed PBMC from 8 study participants and 5 AZD1402 non-exposed donors. PBMC were incubated with vehicle control or PP1-5 for a period 5 days and cytokine/cytolytic molecule secretion quantified by bead array. Bars show mean ± SD; points are individual donors. Colour coding: red = drug-reactive, blue = non-reactive, grey = AZD1402 non-exposed. Dotted horizontal lines indicate assay LLoQ/ULoQ where shown. Differences between peptide treatments were analysed by two-way ANOVA. Comparisons of peptide pools versus unstimulated control used Dunnett’s test; where significance is denoted by *, *p* < 0.05; **, *p* < 0.01; ***, *p* < 0.001; ****, p ,0.0001. ULoQ- upper limit of quantification; LLoQ- lower limit of quantification

All 5 peptide pools stimulated cytokine secretion, as detected by global multiplex analysis, from PBMCs across all cohorts, irrespective of prior exposure to AZD1402 or adverse event manifestation. A range of T-cell cytokines were detected including, but not restricted to, IFN-γ, TNF-α, MIP-1β and IL-6 (Fig. 2a). Interestingly, Th2 cytokines such as IL-4 and IL-13 were only detected at levels close to unstimulated controls (IL-4) or lower limits of qualification (IL-13), although statistically significant IL-4 secretion was observed to all 5 peptide pools. Peptide stimulation did not increase the levels of EGF, FGF-2, Eotaxin, Fit-3 L, fractalkine, MCP-3, IP-10, VEGF, IL-8, MCP-1, IL-15, IL-17, IL-9 and IL-3 above those observed in the untreated control. No statistically significant differences were observed between the donor groups (drug-reactive, non-reactive, non-exposed), other than the unstimulated controls of IL-4 (Supplemental Fig. 2). Principal Component Analysis (PCA) plots were created to visualise the overall differences in cytokine responses between peptide treatments and donor groups (Supplementary Fig. 3). Although the control group could be distinguished from the peptide-treated groups, the cytokine responses did not differ significantly among the various peptide stimulations (Supplementary Fig. 3). No difference in cytokine secretion was observed when peptide pool stimulated PBMC from pharmacokinetic study participants and AZD1402-naive donors were compared, although donor 1118 appeared to be an anomaly with a greater response than other donors (Supplementary Fig. 4). Given the strong IFN-γ and Th1-like cytokine secretion profile detected from the PP1-5-treated PBMC, analysis was performed to measure the secretion of cytolytic molecules traditionally secreted by CD8 cells, granzyme B and perforin, from study participant PBMC. PBMC exposed to PP1-5 secreted both cytolytic molecules, when compared with vehicle control values although no correlation between AZD1402-exposed or non-exposed individuals, or those displaying an adverse event in the clinical study, could be detected (Fig. 2b).

### IFN-γ secretion from drug-naïve PBMC is associated with highly modified regions of AZD1402

**Fig. 3:**
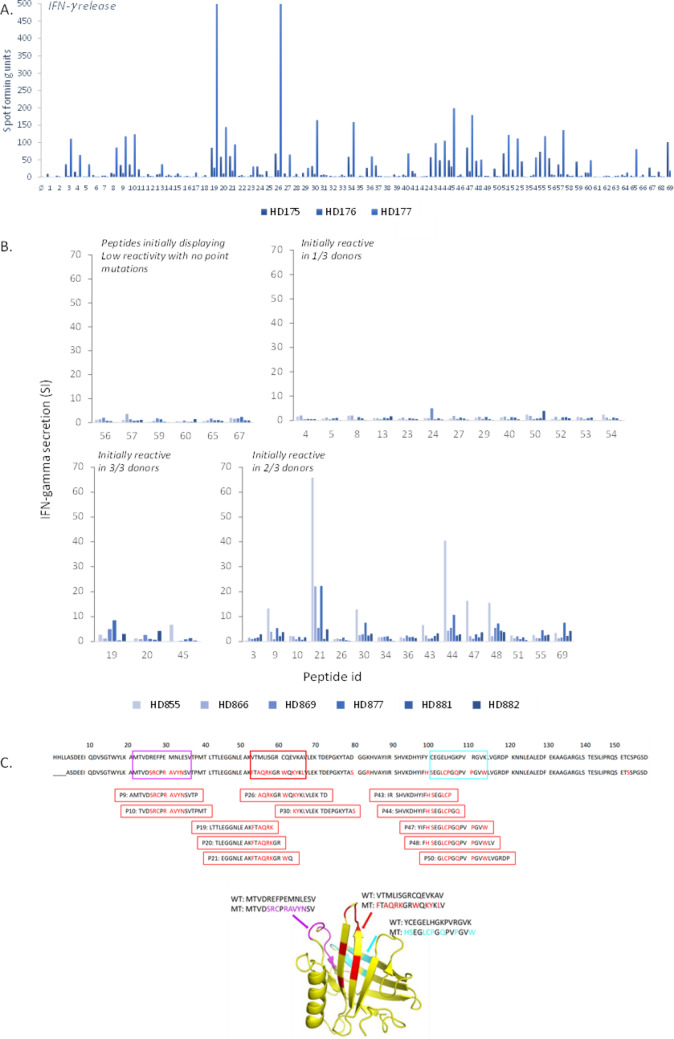
Characterisation of AZD1402 stimulatory peptides. **A** IFN-γ secretion from drug-naïve individual PBMC cultured with individual AZD1402-derived peptides (10µM) for 48 h. Data are presented as spot forming units. **B** Peptides were grouped according to stimulatory properties (derived from data in 4 A) and IFN-γ secretion was measured following stimulation of PBMC from 5–6 additional blood donors. PBMC were incubated in the presence of individual peptides (10µM) for 48 h. Data are expressed as a stimulation index (spot forming units in test incubations with peptide/spot forming units in medium control). **C** Comparison between tear lipocalin (top) and AZD1402 (bottom) amino acid sequences with point mutations highlighted in red. The amino acid sequences highlighted in coloured boxes show AZD1402 sequences that activated donor PBMC. AZD1402 stimulatory epitopes are outlined red rectangular boxes and were pooled to create an activating peptide pool (Primary Peptide Pool, PPP). Scheme displays the position of the 3 “hot spot” immunogenic regions within the 3D structure of AZD1402

IFN-γ secretion was observed when PBMC were exposed to multiple peptides spanning the structure of AZD1402. Figure 3a summarises the results from 3 drug-naive donors, with high levels of IFN-γ secreted in response to some peptides from 2 of the 3 donors. PBMC activation and IFN-γ secretion appeared associated with certain regions of the molecule as defined by overlapping peptides for that region, suggesting that each peptide comprises a common epitope for T-cell activation.

From the above experiment, peptides were divided into 4 categories: Group 1, peptides with no point mutations but seen to stimulate (often low levels) IFN-γ secretion from PBMC from at least one donor; Group 2, peptides with point mutations seen to stimulate (often low levels) IFN-γ secretion from PBMC from one donor; Group 3, peptides with point mutations seen to stimulate IFN-γ secretion from PBMC from 2 out of 3 donors; and Group 4, peptides with point mutations seen to stimulate IFN-γ secretion from PBMC from all drug-naïve donors tested. Peptides from each of these groups were cultured with PBMC from 5 to 6 additional AZD1402 non-exposed donors and IFN-γ secretion expressed as a stimulation index to allow for simpler donor-to-donor comparison. Most peptides in groups 3 and 4 stimulated IFN-γ secretion from PBMC; however, IFN-γ secretion following PBMC culture with peptides from groups 1 and 2 was not detected (Fig. 3b). Based on the above data, a PBMC stimulatory pool of 12 peptides named as the primary peptide pool (PPP) was generated (Fig. 3c shows the peptide sequences and location within AZD1402).

### PBMC are not activated with tear lipocalin-derived peptides

**Fig. 4:**
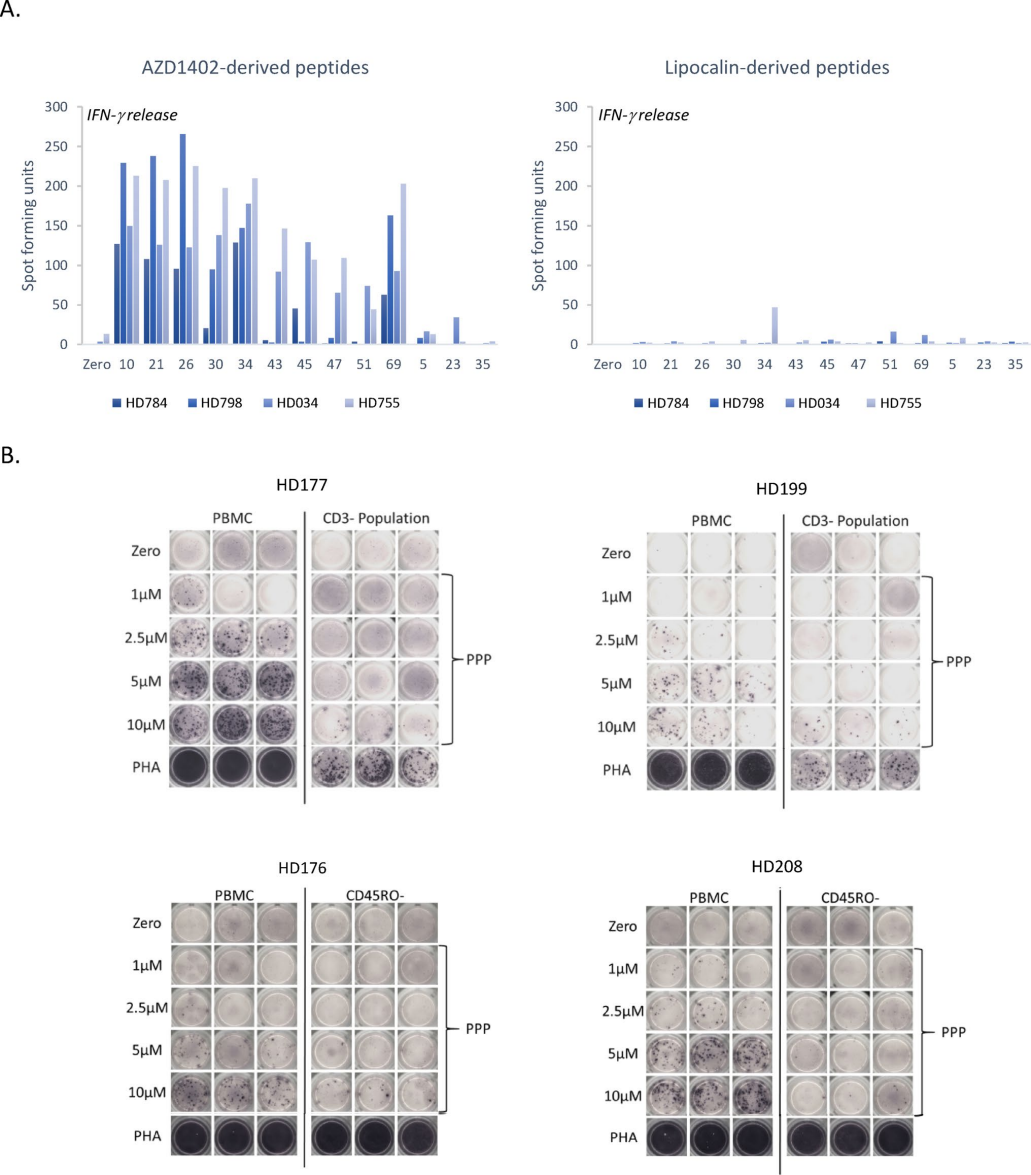
Activation of T-cells by AZD1402-derived peptides is not observed with tear lipocalin-derived matching peptides. **A** IFN-γ ELISpot with PBMC from AZD1402 non-exposed donors. PBMCs from 4 individuals were incubated with stimulatory AZD1402-derived peptides or tear lipocalin equivalents (10µM) for 48 h with R9 medium as a negative control. Peptides 23 and 35, which contained multiple point mutations, but displayed low levels of reactivity were used as additional controls. **B** Depletion of memory T-cells from PBMC abrogates the PPP-specific IFN-γ secretion, as detected by ELISpot spot formation. PBMC, CD3-depleted PBMC (top) or CD45RO-depleted PBMC (bottom) from 2 AZD1402 non-exposed donors were incubated with titrated concentrations of the PPP for 48 h with R9 medium as a negative control and PHA as a positive control T-cell stimulant

The above analyses indicated that all AZD1402-derived peptides stimulatory to PBMC contained at least one, and often more, amino acid point mutation. To confirm that these point mutations were responsible for the observed PBMC reactivity, experiments were conducted comparing AZD1402 stimulatory peptides alongside native tear lipocalin sequence equivalents. A selection of 10 AZD1402-derived peptides stimulated the secretion of IFN-γ from PBMC from 4 drug-naïve donors, whereas little or no secretion was observed with corresponding peptides derived from tear lipocalin sequences (Fig. 4a). Peptides 5, 23 and 35, all containing several point mutations and previously shown to stimulate little cytokine secretion, were included as controls.

### IFN-γ is secreted from T cells

To determine whether the observed increases in PBMC proliferation and IFN-γ secretion were a function of T-cell activation, experiments were conducted to measure PPP-specific IFN-γ secretion from CD3 + and memory T-cell-depleted PBMC populations. A concentration-dependent secretion of IFN-γ was observed with PBMC from AZD1402-naive individuals exposed to the PPP. In contrast, the levels of IFN-γ detected with the PPP-treated CD3-depleted PBMC was similar to that observed in vehicle control wells (Fig. 4b). Furthermore, depletion of the memory T cell subset alone proved sufficient to return the secretion of IFN-γ to baseline control levels. These findings clearly implicate the T memory cell population as the primary responding cell type.

### Characterisation of AZD1402 and AZD1402 peptide-responsive T-cell clones

**Fig. 5:**
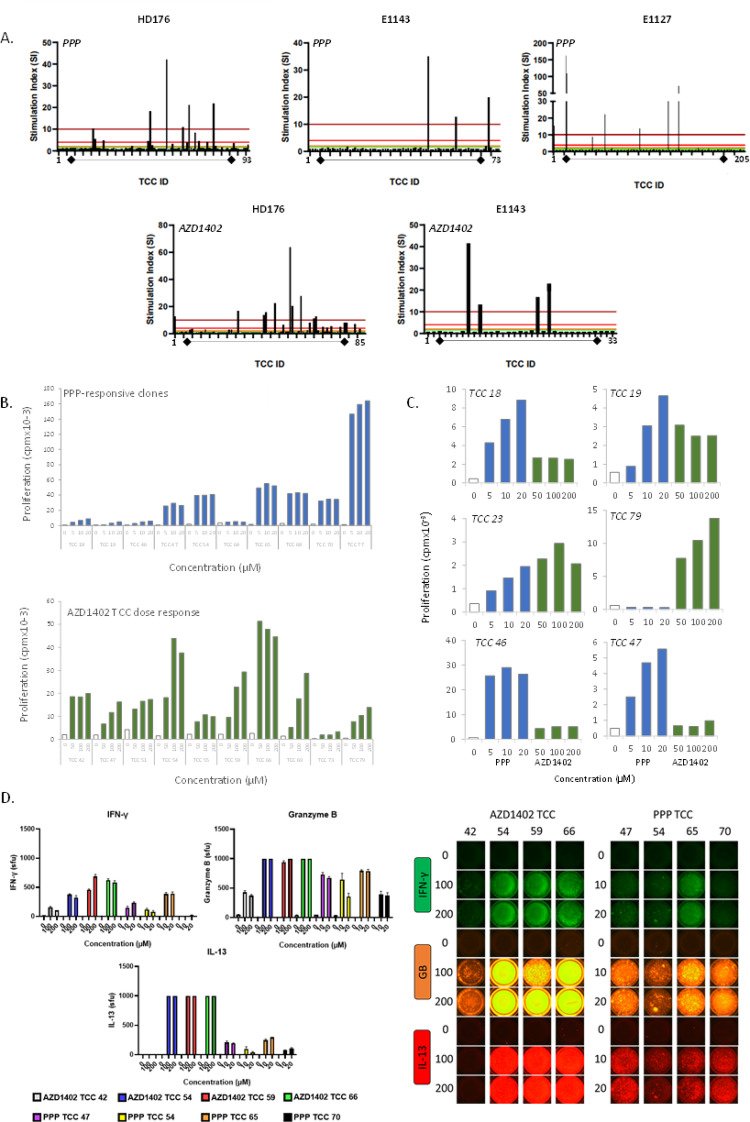
Generation and characterisation of PPP and AZD1402-responsive T-cell clones. **A** T-cell clones were generated from a AZD1402 non-exposed donor and study participants E1127 and E1143. PBMC were cultured with either the PPP or AZD1402 to generate T-cell lines. The T-cell lines were then cloned by serial dilution and repetitive mitogen expansion. T-cell clones were then cultured with irradiated autologous EBV-transformed B-cells and either AZD1402 or PPP for 48 h and [^3^H] thymidine added for an additional 16 h to assess proliferation. Coloured lines indicate weak (green), mid (amber) and strongly reactive clones (red shades). All clones with an SI of 2 or above were expanded for further characterisation. **B** Concentration-dependent proliferative response of representative PPP- or AZD1402-derived T-cell clones stimulated with PPP or AZD1402, respectively. T-cell clones were cultured with irradiated EBV-transformed B-cells and titrated concentrations of PPP or AZD1402 or PPP for 48 h. **C** Cross reactivity of representative T-cell clones. T-cell clones were cultured with irradiated EBV-transformed B-cells and titrated concentrations of AZD1402 and PPP for 48 h **B**,** C** with [^3^H] thymidine added for an additional 16 h to assess proliferation. **D** IFN-γ, IL-13 and granzyme B secretion from AZD1402- and PPP-treated T-cell clones. Clones were incubated with EBV transformed B-cells in a low fluorescent ELISpot plate, pre-coated for IFN-γ, IL-13 and granzyme B in the presence of a titrated concentrations of AZD1402 or PPP for 48 h with R9 medium as a negative control. Data displayed as spot forming units quantified per well and as Fluorospot image. For the Fluorospot readout, an arbitrary value of 1000 counts was recorded when the reader reached saturation (“too numerous spots to count”)

T-cell clones were generated from 3 donors (drug-naive healthy donor HD176, and study participants E1127 and E1143 with no adverse events). PBMC were exposed to PPP for 14 days to enrich the number of responsive T-cells. Clones were tested for antigen-specific proliferation using EBV-transformed B-cells as antigen presenting cells and either the PPP or AZD1402 full protein. AZD1402 and PPP-responsive T-cell clones were isolated from each participant, with 31 and 36 clones displaying proliferative responses towards AZD1402 and PPP, respectively (stimulation index of 2 or above; Fig. 5a). Of these clones, greater than 70% were found to proliferate in a concentration-dependent manner following further expansion (Fig. 5b, concentration-response characteristics of 20 representative clones). Sixteen out of 40 clones were cross-reactive and proliferated in the presence of PPP and AZD1402; the remaining clones were activated by either PPP or AZD1402 alone (Fig. 5c, 6 representative clones). T-cell phenotype was defined by flow cytometry and revealed that all clones obtained from the AZD1402 naïve donor were CD4+ (data not shown). From the 16 clones derived from study participants, 12 were CD4+, while 4 expressed CD8 (2 from each participant).

Secretion of IFN-γ, IL-13 and granzyme B from 8 selected clones (4 PPP- and 4 AZD1402-responsive) were assessed via fluorospot. Varying levels of IFN-γ and granzyme B were secreted from all clones exposed to AZD1402 or the PPP, irrespective of previous AZD1402 exposure (Fig. 5d). IL-13 was secreted from 7 of 8 T-cell clones.

To investigate the HLA restriction of the response, class I and II MHC molecules were blocked during the cultures using specific antibodies. MHC class II block led to abrogation of both the PPP and AZD1402-specific proliferative responses in CD4 + T-cell clones, with MHC class I block having little or no effect (Supplementary Fig. 5). In contrast, with the single CD8 + clone available for testing, MHC class I block reduced the proliferative response whilst MHC class II block had little impact. IgG Isotype controls were included in the assay, with no effect on the PPP or AZD1402-dependent T-cell activation (data not shown).

### Assessment of the proliferative response of AZD1402- and PPP-responsive T-cell clones to tear lipocalin and lipocalin-derived peptides

**Fig. 6:**
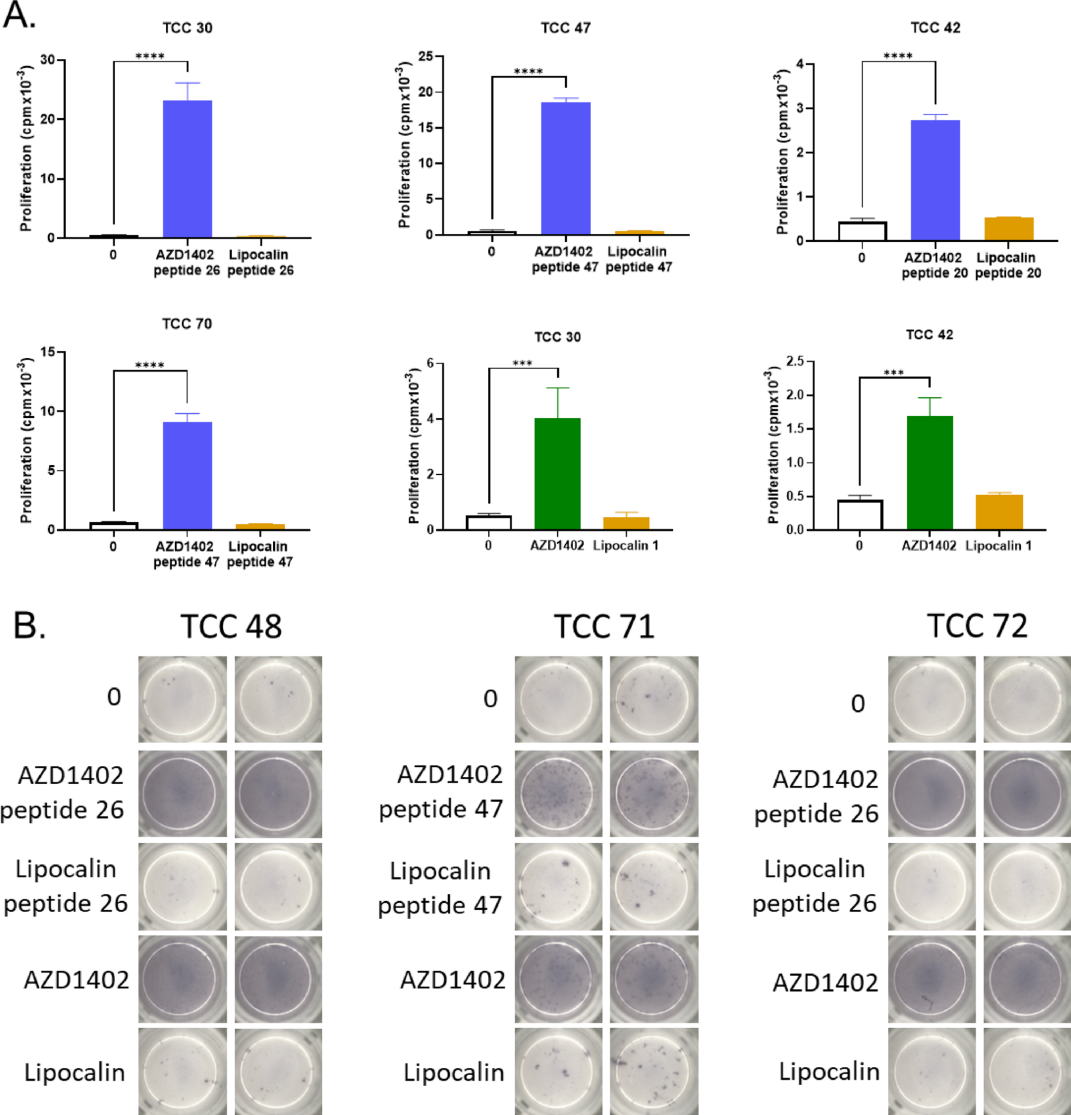
Activation of T-cell clones with AZD1402- and AZD1402-derived peptide is not observed with lipocalin or lipocalin-derived peptides. T-cell clones were cultured with irradiated EBV-transformed B-cells and AZD1402 (green) or AZD1402-derived peptides (blue) and lipocalin equivalents (yellow) for 48 h. **A** Proliferation and **B** IFN-γ secretion were assessed as markers of T-cell activation. The data was analysed by Two-way ANOVA and * denotes statistical significance P* <0.05, ** *P*<0.01, *** *P*<0.001, **** *P*<0.0001

To confirm whether the point mutations in AZD1402 that afford the molecule target binding capabilities were responsible for the observed T-cell responses, experiments were conducted with 9 T-cell clones comparing AZD1402 and AZD1402-stimulatory peptides with tear lipocalin and native peptide equivalents. Clones displaying proliferative responses against AZD1402 (*n* = 2) or AZD1402 peptides P20, P26 and P47 (*n* = 4) were not activated with tear lipocalin or the equivalent tear lipocalin-derived peptides (Fig. 6a). Three AZD1402 and AZD1402 peptide cross-reactive clones were used in IFN-γ ELISpot assays. Clones were found to secrete IFN-γ in the presence of AZD1402 and peptides P26 or 47, but not with tear lipocalin or the corresponding lipocalin-derived peptides (Fig. 6b).

### Identification of key areas of AZD1402 T-cell reactivity through assessment of overlapping AZD1402 peptides

**Fig. 7:**
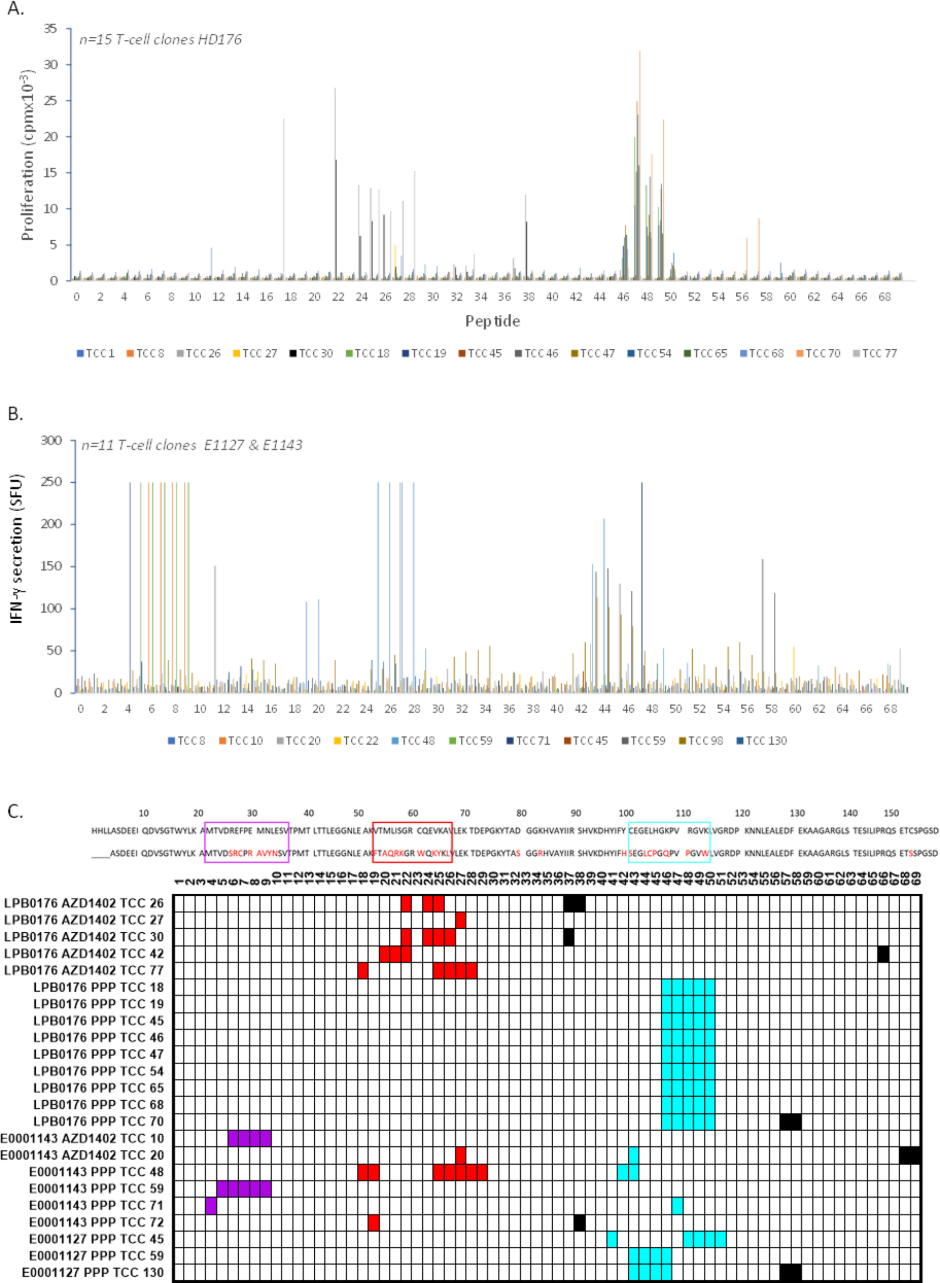
Characterisation of AZD1402 stimulatory peptides. T-cell clones from a non-exposed individual **A**; *n* = 15) and AZD1402 study participants **B**; *n* = 11) were cultured with EBV-transformed B-cells and individual AZD1402-derived peptides (10µM) for 48 h. **A** Proliferation or **B** IFN-γ secretion denote T-cell activation. For the ELISpot readout an arbitrary value of 250 counts was assigned when the reader saturated (“too numerous spots to count”). **C** Epitope map displaying stimulatory peptides for individual clones derived from the 3 donors. Three hotspot regions of immunogenicity containing sites of extensive point mutation are colour coded

Individual AZD1402 peptides were cultured with T-cell clones from AZD1402-exposed participants (*n* = 11), and an AZD1402-naive individuals (*n* = 15) in the presence of autologous antigen presenting cells to establish an epitope map of the key regions of reactivity for AZD1402 (Fig. 7a). Clones from the non-exposed donor HD-176 were activated with peptides in 2 “hotspot regions” spanning peptides 18–28 and 46–50. Eight clones from study participant E1143 were activated with peptides in three hotspot regions spanning peptides 6–10, 18–28 and 42–50. Noticeably, fewer clones derived from study participant E1143 were activated with peptides in positions 42–50, when compared with results obtained for naïve donor HD176 clones. Three clones from study participant E1127 were expanded in sufficient numbers to perform epitope mapping. These clones were activated with peptides from the hotspot region spanning peptides 42–50 (Fig. 7b). A heat map of AZD1402 T-cell stimulatory peptides (Fig. 7c) with the positioning of the reactive epitopes aligned to the 3D structure of AZD1402 (Fig. 3c).

## Discussion

AZD1402 is an anticalin modality based on the human tear lipocalin sequence comprising 28 amino acid point mutations located for the most part across 4 loop structures (IL4-Rα-targeting domains) to enhance target selectivity and specificity. A small AZD1402 clinical bridging study conducted in 2019 resulted in the observation of various potential AZD1402-related adverse events in 5 of 18 trial participants several hours after a third exposure (doses administered at least 5 days apart). Trial participants with adverse events were also tested weakly positive for ADAs against AZD1402 following three exposures. Thus, the objectives of this analysis were to investigate whether exposure to AZD1402 in vitro could stimulate a T-cell recall response in the study participants and, if so, to characterise the phenotype and function of the AZD1402-responsive T-cells. Since in vitro experiments with biologic molecules often underestimate the potential for immunogenicity, overlapping peptides (stepped by 2aa) spanning the AZD1402 structure were created and assessed, alongside the formulation excipients and AZD1402 full protein, in PBMC assays to measure antigen-specific proliferative responses and IFN-γ secretion. A similar approach has previously been shown to be effective to explore the immunogenicity of therapeutic proteins such as infliximab and rituximab (Hamze et al. 2017).

PBMC from clinical study participants (with and without adverse events) displayed reactivity against all 5 peptide pools, whereas weaker responses were detected with the full protein AZD1402. Luminex analysis revealed that the peptide pools stimulated the secretion of high levels of Th1 cytokines (e.g., IFN-γ, TNF-α) and cytolytic molecules (perforin, granzyme B), which if generated in patients could potentially contribute to adverse events. In contrast, Th2 cytokine secretion (e.g., IL-4, IL-13) was not elevated, or only marginally elevated, over control values. Secretion of IFN-γ was all but abolished when total T-cell or memory T-cell populations were removed from the assessments. Collectively, these data indicate that AZD1402-derived peptides activate memory T-cells that circulate in almost all exposed individuals, irrespective of whether an adverse clinical observation was noted in the clinical study.

To explore whether the observed AZD1402-specific activation of study participant PBMC was associated with AZD1402 exposure, experiments were conducted using PBMC from AZD1402-naïve donors. Interestingly, similar peptide pool-specific proliferative responses and cytokine secretion were detected with PBMC from AZD1402 non-exposed donors. This could indicate that to some extent a memory response directed against an unidentified antigen previously encountered by most individuals tested may be responsible for the T-cell activation observed. The National Centre for Biotechnology Information BLASTp sequence homology tool and immune epitope database were used to assess homology of the AZD1402 full protein and derived peptides to known immunogenic antigens. We were unable to identify significant levels of homology to other immunogenic antigens other than lipocalin superfamily proteins. All the shared sequences identified were derived from regions of AZD1402 with no modifications and no subsequent immunogenicity in our assays.

Furthermore, it appeared that peptides located in “hot spot” regions of AZD1402 were repeatedly associated with PBMC proliferation and cytokine secretion in the majority of donors. These hot spots regions were in 3 out of 4 loop structures of AZD1402 that each contain areas of extensive amino acid point mutations, the importance of which in AZD1402 T-cell reactivity was confirmed through synthesis and assessment of equivalent peptides found in tear lipocalin. Equivalent peptides derived from the native tear lipocalin sequence did not activate T-cells to proliferate or secrete IFN-γ. The 12 most consistent stimulatory AZD1402 peptides were selected to establish a primary peptide pool (PPP), which was further utilised in the generation and characterisation of T-cell clones.

AZD1402 and PPP T-cell clones were generated from individual AZD1402 pharmacokinetic study participants, alongside an AZD1402 non-exposed donor. CD4 + T-cell clones displaying proliferative responses against AZD1402 full protein and the PPP were detected in all three donors. Although clones from the donors were stimulated to proliferate and secrete cytokines in the presence of different AZD1402-derived peptides, all reactive peptides mapped to the “hot spot” regions within the AZD1402 protein. Donor-to-donor variation in T-cell epitopes likely relates to the HLA alleles expressed by each participant and binding of the peptides to different MHC class II proteins. As noted with the PBMC experiments, proliferation and cytokine secretion from T-cell clones was not detected with equivalent native tear lipocalin sequence-derived peptides, demonstrating an AZD1402-specific response and providing evidence that the recall responses detected are not directed towards the scaffold tear lipocalin molecule itself. Several T-cell clones were activated by both AZD1402 and the PPP, illustrating that antigen processing from the full-length AZD1402 can occur in the assay. However, other clones displayed a highly specific response to either the full protein or PPP. This indicates that (i) certain AZD1402-responsive clones are activated by additional AZD1402-derived peptides, perhaps containing a different number of amino acids, formed through processing of the protein, and (ii) several PPP-responsive clones are activated by peptides not generated naturally though processing of AZD1402, or not generated in sufficient quantities by the antigen presenting cells available in the culture.

Introduction of amino acid substitutions into any human protein runs the risk of generating novel non-self, immunogenic epitopes that can be presented by MHC molecules and generate *de novo* T-cell responses. Alternatively, as seems to be the case with AZD1402, novel peptide sequences may have sufficient similarity to previously encountered non-self peptides to allow activation of pre-existing memory T-cells. This concept of molecular mimicry was first described in the 1960s, where it was used to define similarities between peptides expressed by pathogens and human cells (Damian 1965, 1987). Several reports describe sequence homologies between pathogen-derived peptides and peptides displayed by human cells (Herrath and Oldstone 1996; Johnson and Jiang 2023) with the induction of cross-reactive immune responses.

It is intriguing to consider why approximately 30% of study participants reacted adversely to AZD1402, whilst the remainder were free from clinical observations over all 3 doses of the molecule. One possibility is that treatment in tolerant participants might simply have been stopped before the observed T-cell response translated into an adverse event. However, it is also possible that some study participants may tolerate AZD1402 due to a favourable immune regulatory status at the time of drug exposure. A complex regulatory network including Tregs (Benamar et al. 2023; Georgiev et al. 2024), co-inhibitory receptor ligand interactions [e.g., PD-L1, CTLA4] (Ahmadzadeh et al. 2009; Sakuishi et al. 2010) and regulatory cytokines [e.g., IL-10, TGF-β] (Talaat et al. 2015; Thunberg et al. 2007) exists that maintains a threshold level of natural immunological tolerance. Such thresholds differ amongst individuals, and may differ in the same individual throughout their life, particularly during periods of exposure to infectious agents and disease. Therapeutic protein specific T-cells are often considered a precursor for the induction of anti-drug antibodies (ADAs) in vivo (Vaisman-Mentesh et al. 2020; Jawa et al. 2020). However, the presence of therapeutic protein-specific T-cells and/or ADAs alone does not necessarily result in a clinically relevant adverse event. Induction of tolerance against a specific biologic will influence whether a protein is tolerated both in vivo and in vitro. Tolerogenic mechanisms, whether patient specific or attributable to the therapeutic protein itself can ameliorate the induction of effector T-cell responses and associated T-cell mediated toxicities. Perturbation of these mechanisms can lead to adverse events (Naisbitt et al. 2020). This is a plausible reason as to why individuals/patient groups with HIV infection or cystic fibrosis are particularly susceptible to the development of drug-induced immune adverse events (Carr and Cooper 1995; Parmar 2005; Pleasants et al. 1994). Lessons can also be learned from the field of immune-oncology, where the incidence of immune-related adverse event cases to concomitant medications is increased when co-inhibitory molecule signalling is blocked via immune checkpoint inhibitor therapy (Ford et al. 2018; Naisbitt et al. 2020; Hammond et al. 2022). When considering AZD1402 and future molecules adapted from endogenous proteins in general, it is plausible that adverse immune events may develop at any time during long-term drug treatment if the immune context and regulatory threshold is disrupted by an unrelated event. Furthermore, with inhaled biologics targeting lung diseases such as asthma and COPD, immunosuppressive comedications medications given as standard of care may impact the risk of overt immune activation.

A parallel can be drawn between the clinical and laboratory findings with AZD1402 and abacavir hypersensitivity. With abacavir hypersensitivity, drug-responsive T-cells are detectable in 100% of drug-exposed and drug-naive individuals expressing HLA-B*57:01, despite the fact that only 50% of exposed patients develop adverse events (Bell et al. 2013; Schnyder et al. 2013; Adam et al. 2014; Thomson et al. 2020). These data clearly indicate that activation of T-cells with abacavir is the key molecular initiating event; however, the presence of abacavir-responsive T-cells does not always translate into a clinically relevant adverse event.

In conclusion, the AZD1402 structure contains multiple T-cell epitopes located in “hotspot” regions of the molecule. Similar T-cell epitopes are not found in tear lipocalin. These data describe a potential immunogenicity risk associated with AZD1402 and provide a practical framework which could be deployed to explore the potential immunogenicity of putative therapeutic proteins.

## Supplementary Information

Below is the link to the electronic supplementary material.

Supplementary Material 1

## Data Availability

All data is available from authors upon request.

## Abbreviations

PBMC: Peripheral blood mononuclear cells
SI: Stimulation index
PP: Peptide pool
PPP: Primary peptide pool
EBV: Epstein-barr virus
PHA: Phytohemagglutinin
HLA: Human leukocyte antigen
