# Supplementary material for: The immunogenic potential of AZD1402 (Elarekibep) T-cell epitopes in healthy volunteers and drug-exposed clinical trial participants
Source: Arch Toxicol. 2026 Apr 28;100(8):3535–52. doi: 10.1007/s00204-026-04403-1 (PMC13379463; doi:10.1007/s00204-026-04403-1)
Supplement: Supplementary file 1 — Supplementary Material 1 [file 204_2026_4403_MOESM1_ESM.docx]

**Supplementary Materials:**

**Supplementary Fig. 1.** Assessment of test substance-induced study subject and non-exposed individual PBMC proliferation.

**Supplemental Fig. 2.** Comparison of cytokine secretion from three different donor groups (drug-reactive, non-reactive, non-exposed).

**Supplementary Fig. 3**. Principal Component Analysis (PCA) of cytokine responses in PBMCs exposed to peptide pools – categorised by treatment.

**Supplementary Fig. 4.** Principal Component Analysis (PCA) of cytokine responses in PBMCs exposed to peptide pools – categorised by donor profile.

**Supplementary Fig. 5.** MHC-restricted activation of T-cell clones with AZD1402 or AZD1402-derived peptides.

**Supplementary Table 1.** AZD1402 Peptide pool allocation.

**Supplementary Fig. 1. Assessment of test substance-induced study subject and non-exposed individual PBMC proliferation.** (A) Amino acid sequence comparison of tear lipocalin (top) and AZD1402 (bottom) with amino acid substitutions illustrated in red. PBMC from study subjects (B) and AZD1402 non-exposed individuals (C) were incubated with the PP1-5, excipient 1 or 2, or AZD1402 full protein for a period of 5 days with R9 medium as a negative control. [^3^H]thymidine was added for an additional 16 hours, and proliferation was assessed by scintillation counting. Individuals in red colour coding indicates adverse observations in the clinical trial, blue colour coding denotes individuals with no adverse reactions in the clinical trial. Data shown are means from duplicate wells.

**Supplemental Fig. 2. Cytokine and cytolytic molecule secretion from peptide pool-exposed PBMC from 8 study participants and 5 AZD1402 non-exposed donors.** PBMC were incubated with vehicle control or PP1-5 for a period 5 days and cytokine/cytolytic molecule secretion quantified by bead array. Bars show mean ± SD; points are individual donors. Colour coding: red = drug‑reactive, blue = non‑reactive, grey = AZD1402 non‑exposed. Dotted horizontal lines indicate assay LLoQ/ULoQ where shown. Pairwise differences between donor groups were tested with Tukey’s multiple comparisons (Šidák used for perforin and granzyme B), where significance is shown as *, p<0.05 and **, p< 0.01. ULoQ- upper limit of quantification; LLoQ- lower limit of quantification.


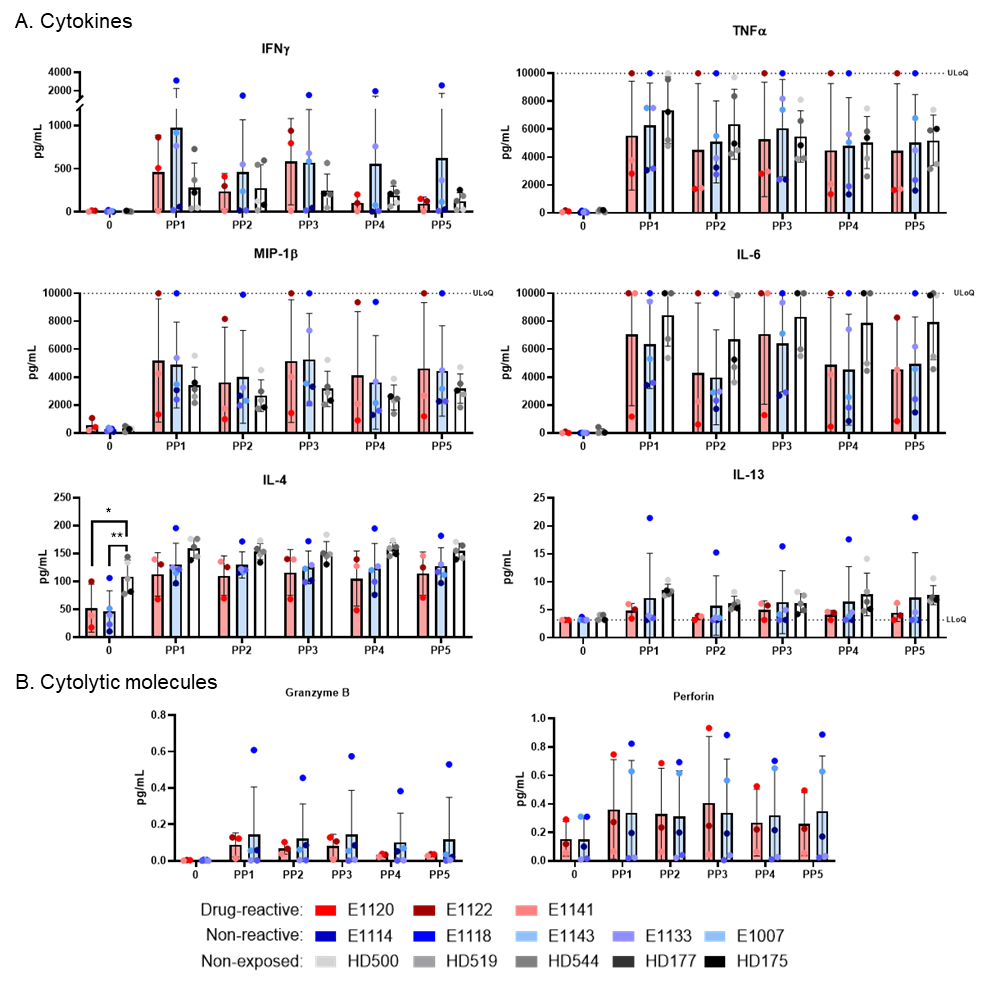

**Supplementary Fig. 3. Principal Component Analysis (PCA) of cytokine responses in PBMCs exposed to peptide pools – categorised by treatment.** PCA plot illustrating the first two principal components (Dim1 and Dim2) derived from cytokine induction in PBMCs exposed to peptide pools 1-5, or media control. Dim1 accounts for 55.7% of the variance, while Dim2 accounts for 13.3%. Data points represent individual responses to treatments, categorised by treatment: peptide pool 1 (blue triangle), 2 (green +), 3 (Yellow X), 4 (purple diamond), 5 (blue inverse triangle), and control treatment (red circle), and media control (no cells, brown square with x). Media represents conditions with no cells. Ellipses represent confidence intervals calculated using a T distribution around the data points for each group, illustrating the variability and general response pattern to each peptide pool or control.

**Supplementary Fig. 4. Principal Component Analysis (PCA) of cytokine responses in PBMCs exposed to peptide pools – categorised by donor profile.** PCA plot illustrating the first two principal components (Dim1 and Dim2) derived from cytokine induction in PBMCs exposed to peptide pools 1-5, or media control. Dim1 accounts for 55.7% of the variance, while Dim2 accounts for 13.3%. Data points represent individual responses to treatments, categorised by donor profile: drug reactive (red circles), drug tolerant (blue triangles), naïve (green squares), and media control (purple crosses). Media represents conditions with no cells. Ellipses represent confidence intervals calculated using a T distribution around the data points for each group, illustrating the variability and general response pattern of each donor profile.

**
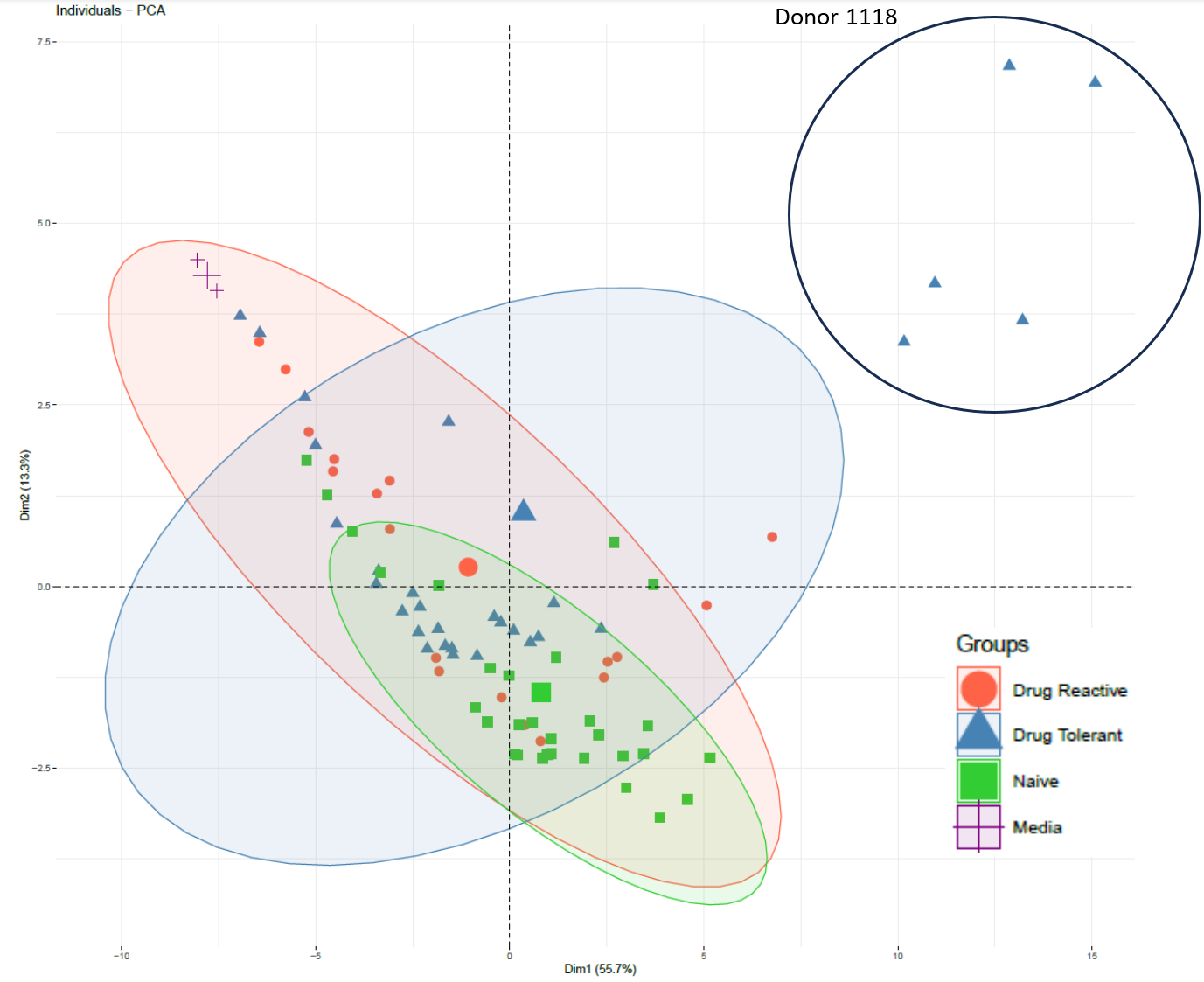
**

**Supplementary Fig. 4. MHC-restricted activation of T-cell clones with AZD1402 or AZD1402-derived peptides.** AZD1402 and PPP-derived T-cell clones were cultured with AZD1402 or PPP and autologous EBV-transformed B-cells for 48 h in the presence or absence of MHC blocking antibodies). Blocking antibodies were added to the culture assay 2 hours prior to AZD1402/PPP. (A) [^3^H]thymidine was then added for an additional 16 hours, and proliferation assessed. (B) IFN-γ secretion was characterised by ELIspot.

**Supplementary Table 1. AZD1402 Peptide pool allocation**

| **Peptide Pool 1** | | **Peptide Pool 2** | | **Peptide Pool 3** | | **Peptide Pool 4** | | **Peptide Pool 5** | |
| --- | --- | --- | --- | --- | --- | --- | --- | --- | --- |
| **No.** | **Sequence** | **No.** | **Sequence** | **No.** | **Sequence** | **No.** | **Sequence** | **No.** | **Sequence** |
| 1 | ASDEEIQDVSGTWYLKAM | 13 | PRAVYNSVTPMTLTTLEG | 29 | WQKYKLVLEKTDEPGKYT | 44 | SHVKDHYIFHSEGLCPGQ | 57 | GRDPKNNLEALEDFEKAA |
| 2 | DEEIQDVSGTWYLKAMTV | 14 | AVYNSVTPMTLTTLEGGN | 30 | KYKLVLEKTDEPGKYTAS | 45 | VKDHYIFHSEGLCPGQPV | 58 | DPKNNLEALEDFEKAAGA |
| 3 | EIQDVSGTWYLKAMTVDS | 15 | YNSVTPMTLTTLEGGNLE | 31 | KLVLEKTDEPGKYTASGG | 46 | DHYIFHSEGLCPGQPVPG | 59 | KNNLEALEDFEKAAGARG |
| 4 | QDVSGTWYLKAMTVDSRC | 16 | SVTPMTLTTLEGGNLEAK | 32 | VLEKTDEPGKYTASGGRH | 47 | YIFHSEGLCPGQPVPGVW | 60 | NLEALEDFEKAAGARGLS |
| 5 | VSGTWYLKAMTVDSRCPR | 17 | TPMTLTTLEGGNLEAKFT | 33 | EKTDEPGKYTASGGRHVA | 48 | FHSEGLCPGQPVPGVWLV | 61 | EALEDFEKAAGARGLSTE |
| 6 | GTWYLKAMTVDSRCPRAV | 18 | MTLTTLEGGNLEAKFTAQ | 34 | TDEPGKYTASGGRHVAYI | 49 | SEGLCPGQPVPGVWLVGR | 62 | LEDFEKAAGARGLSTESI |
| 7 | WYLKAMTVDSRCPRAVYN | 19 | LTTLEGGNLEAKFTAQRK | 35 | EPGKYTASGGRHVAYIIR | 50 | GLCPGQPVPGVWLVGRDP | 63 | DFEKAAGARGLSTESILI |
| 8 | LKAMTVDSRCPRAVYNSV | 20 | TLEGGNLEAKFTAQRKGR | 36 | GKYTASGGRHVAYIIRSH | 51 | CPGQPVPGVWLVGRDPKN | 64 | EKAAGARGLSTESILIPR |
| 9 | AMTVDSRCPRAVYNSVTP | 21 | EGGNLEAKFTAQRKGRWQ | 37 | YTASGGRHVAYIIRSHVK | 52 | GQPVPGVWLVGRDPKNNL | 65 | AAGARGLSTESILIPRQS |
| 10 | TVDSRCPRAVYNSVTPMT | 22 | GNLEAKFTAQRKGRWQKY | 38 | ASGGRHVAYIIRSHVKDH | 53 | PVPGVWLVGRDPKNNLEA | 66 | GARGLSTESILIPRQSET |
| 11 | DSRCPRAVYNSVTPMTLT | 23 | LEAKFTAQRKGRWQKYKL | 39 | GGRHVAYIIRSHVKDHYI | 54 | PGVWLVGRDPKNNLEALE | 67 | RGLSTESILIPRQSETSS |
| 12 | RCPRAVYNSVTPMTLTTL | 24 | AKFTAQRKGRWQKYKLVL | 40 | RHVAYIIRSHVKDHYIFH | 55 | VWLVGRDPKNNLEALEDF | 68 | LSTESILIPRQSETSSPG |
|  |  | 25 | FTAQRKGRWQKYKLVLEK | 41 | VAYIIRSHVKDHYIFHSE | 56 | LVGRDPKNNLEALEDFEK | 69 | TESILIPRQSETSSPGSD |
|  |  | 26 | AQRKGRWQKYKLVLEKTD | 42 | YIIRSHVKDHYIFHSEGL |  |  |  |  |
|  |  | 27 | RKGRWQKYKLVLEKTDEP | 43 | IRSHVKDHYIFHSEGLCP |  |  |  |  |
|  |  | 28 | GRWQKYKLVLEKTDEPGK |  |  |  |  |  |  |
